# Preparation of Carriers Based on ZnO Nanoparticles Decorated on Graphene Oxide (GO) Nanosheets for Efficient Immobilization of Lipase from *Candida rugosa*

**DOI:** 10.3390/molecules22071205

**Published:** 2017-07-19

**Authors:** Shan Zhang, Jie Shi, Qianchun Deng, Mingming Zheng, Chuyun Wan, Chang Zheng, Ya Li, Fenghong Huang

**Affiliations:** Hubei Key Laboratory of Lipid Chemistry and Nutrition, Oil Crops and Lipids Process Technology National & Local Joint Engineering Laboratory, Key Laboratory of Oilseeds Processing, Ministry of Agriculture, Oil Crops Research Institute, Chinese Academy of Agricultural Sciences, Wuhan 430062, China; shanzhangvip@sina.com (S.Z.); dengqianchun@caas.cn (Q.D.); zhengmingming@caas.cn (M.Z.); wanchuyun@hotmail.com (C.W.); trump81@163.com (C.Z.); liya@whu.edu.cn (Y.L.)

**Keywords:** graphene oxide, ZnO nanoparticles, immobilized CRL, enzyme activity, reusability

## Abstract

Herein, a promising carrier, graphene oxide (GO) decorated with ZnO nanoparticles, denoted as GO/ZnO composite, has been designed and constructed. This carrier was characterized by X-ray powder diffraction, scanning electron microscopy, Fourier transform infrared spectroscopy and thermogravimetry. Then, *Candida rugosa* lipase (CRL) was immobilized onto the GO-based materials via physical adsorption. Our results indicated that the lipase loading amount on the GO/ZnO composites was about 73.52 mg of protein per g. In the activity assay, the novel immobilized lipase GO/ZnO@CRL, exhibited particularly excellent performance in terms of thermostability and reusability. Within 30 min at 50 °C, the free lipase, GO@CRL and ZnO@CRL had respectively lost 64%, 62% and 41% of their initial activity. However, GO/ZnO@CRL still retained its activity of 63% after 180 min at 50 °C. After reuse of the GO/ZnO@CRL 14 times, 90% of the initial activity can be recovered. Meanwhile, the relative activity of GO@CRL and ZnO@CRL was 28% and 23% under uniform conditions. Hence, GO-decorated ZnO nanoparticles may possess great potential as carriers for immobilizing lipase in a wide range of applications.

## 1. Introduction

Biocatalysts possess excellent properties in terms of efficient catalytic activity, exclusion of undesirable side reactions, operations under mild conditions, and different types of stereoselectivity in some chemical reactions, such as oxidation-reduction [1], hydrolysis [2] and esterification [3]. To date biocatalysts have been widely used in applications including the synthesis of structured lipids [4], synthesis of pharmaceutical intermediates [5] and biofuel production [6]. Among all commercialized enzymes, one of the most popular enzymes, lipases (triacylglycerol ester hydrolases, EC 3.1.1.3) are widely used as adaptable biocatalysts [7,8,9,10] to catalyze a number of reactions making lipases unique biocatalysts in the chemistry, food, agrochemical, biotechnology and pharmaceutical industries due to their excellent regio-, chemo-, and stereoselective properties [11].

However, the practical industrial applications of water-soluble enzymes like lipases are still limited, and some of their drawbacks are their instability, short lifetime and the need for additional efforts to recycle the enzymes from a product mixture [12]. Therefore, to overcome the above disadvantages, the technique of immobilizing lipases on various carriers is considered an effective strategy to improve the stability and facilitate separation from reaction systems [13].

Recently, numerous efforts have been focused on the preparation of lipases in immobilized forms, which involve all sorts of new carrier materials, both inorganic and organic, to improve the catalytic performance [14,15,16,17]. Combined with recent advances in nanotechnology, nanostructured materials with large surface-to-volume ratio such as nanomesoporous materials and carbon nanotubes have been demonstrated as immobilization supports to re-engineer enzyme catalysts for superior stability and activity [18,19].

Graphene, a two-dimensional (2D) honeycomb lattice, offers remarkable chemical stability, mechanical strength, bio-compatibility and lack of toxicity, therefore, it has a great future in many special areas. As a precursor of graphene, graphene oxide (GO) sheets consist of both oxygenated polar domains and aromatic nonpolar domains that endow them with the binary characteristics of a conventional block co-polymer [20]. They also have the proper mechanical strength, good biocompatibility, high specific surface area, scalable manufacture, controllable surface chemistry, and full of functional groups, therefore, the features of GO makes it an ideal carrier for lipase immobilization [21].

So far, very few reports about GO-based supports for lipase immobilization have been published. For instance, Jing and co-workers [22] synthesized a chloropropyl-functionalized graphene oxide decorated with Fe_3_O_4_ nanoparticles for the immobilization of porcine pancreatic lipase (PPL); Mohammadi and co-workers [23] used an isocyanide-based four-component reaction tobimmobilize *Rhizomucor miehei* lipase (RML) on carboxylated multi-wall carbon nanotubes and carboxylated graphene nanosheets; Li and co-workers [24] fabricated three dimensional (3D) magnetic graphene oxide-chitosan (GO/CS) composites with orderly self-assembled magnetite (Fe_3_O_4_) nanoparticles (GO/CS/Fe_3_O_4_) to was immobilize *Candida rugosa* lipase (CRL).

According to recent studies, we can see that covalent binding methods were first selected to immobilize lipase. Because of its ability to rigidify the location of the immobilized enzyme, improved selectivity and stability can be achieved via covalent bonding, however, the most critical point is that the spatial structure of protein can be changed by covalent attachment, leading to damage to the enzyme activity center, thereby reducing the enzyme activity [14,15,24,25,26,27]. Moreover, it is well known that physical adsorption-immobilization via intermolecular interaction, preserves well the structures of both the nanomaterial and the protein, and maximizes the catalytic performance of enzymes. Some enzymes may leach from the carrier, but this drawback can be solved by optimizing the carrier structure.

Zinc oxide (ZnO) nanoparticles have been exploited in many fields such as catalysis, drug delivery and biology, where they have unique ability to promote faster electron transfer between the active site of enzymes and substrates, as a potential material in enzyme engineering, however, very little information about their application in immobilization of lipase can be found [28].

**Scheme 1:**
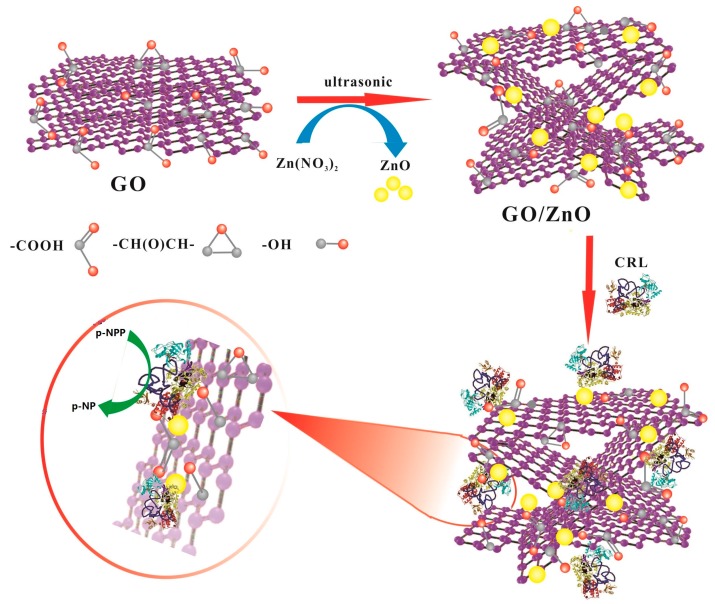
Schematic representation of the formation of GO/ZnO@CRL.

Herein, we report a novel carrier for the immobilization of lipase via intermolecular interactions such as hydrogen bond and/or complementary electrostatic attraction prepared by integrating ZnO nanoparticles with GO. The formation of the GO/ZnO combined supporting material is illustrated in Scheme 1 and the design concept explained as follows: (1) because of the interactions between the layers, GO nanosheets are inclined to agglomerate, which leads to a lower surface area, yet covered ZnO nanoparticles can effectively prevent the agglomeration of GO; (2) GO comprises hydrophilic edges and hydrophobic nanosheets [20]. It has a lot of oxygen-containing groups including hydroxyl, epoxy, carbonyl, and carboxyl groups, so that the functional groups and the large surface area can be retained to interact with the lipase. These groups change the properties of graphene from hydrophobic to strongly hydrophilic, and the existence of negatively charged carboxylate groups can promote binding to proteins via electrostatic interactions. In addition, ZnO nanoparticles are hydrophilic, hence, this complex possesses good dispersibility in water, which makes it suitable to immobilize lipase easily. Furthermore, the hydrophobic parts of GO nanosheets may drive the lipase to open the “lid”, expose the active site, and enhance the catalytic performance, they can also facilitate the dispersion interaction driven binding of enzymes, eliminating the need for chemical modification of the protein prior to surface conjugation [29,30]; (3) for their unusual properties, namely, a high isoelectric point of 9.5, which allows for the immobilization of lipase (*Candida rugosa* lipase: a low isoelectric point of 4.6) through an electrostatic interaction, ZnO nanoparticles were incorporated as a covered functional group here [31]. Moreover, an enhanced surface area can be achieved by the ZnO nanoparticles, allowing for the strong adsorption of lipase, improved catalytic efficiency, chemical stability, biocompatibility and high electron transfer ability [32].

On the basis of the foregoing information, we have designed a novel supporting material, which integrates GO with ZnO nanoparticles, for better enzyme immobilization. *Candida rugosa* lipase (CRL) was selected as a model enzyme in order to assess the potential applicability of GO/ZnO composites as a new carrier. Meanwhile, for comparison purposes, we prepared another two immobilized lipases, GO@CRL and ZnO@CRL, and together with GO/ZnO@CRL, their physical properties, relative activity, stability and reusability were investigated.

## 2. Results and Discussion

### 2.1. Design, Synthesis and Structural Characterization of GO/ZnO Composites

The design rationale is illustrated in Scheme 1. Firstly, graphene oxide (GO) was prepared in a classical way, Hummer’s method, in which plentiful oxygen-containing functional groups can be observed. In addition, after Zn^2+^ was added, in-situ formed ZnO clustered particles decorate and prevent the restacking of GO sheets and the ordered lamellar GO was exfoliated for a shaggier surface [33]. When we immobilized CRL lipase on the obtained supports (GO/ZnO), CRL was randomly distributed on the surface of GO/ZnO. According to the literature, CRL not only interacted with the oxygen-containing functional group on GO sheets through hydrogen bond interactions [20], but also combined with ZnO clustered particles via electrostatic interactions [34]. The two interaction forces successfully guarantee the stability of the immobilized lipase (GO/ZnO@CRL). During the hydrolytic process, the *p*-nitrophenylpalmitate (*p*-NPP) substrate surround GO/ZnO@CRL nanoparticles where the hydrolysis product *p*-nitrophenol (*p*-NP) is produced and the GO/ZnO exfoliated layers provide a high surface area for the mass transfer of *p*-NP product for high catalytic performance.

### 2.2. Materials Characterizations

**Figure 1:**
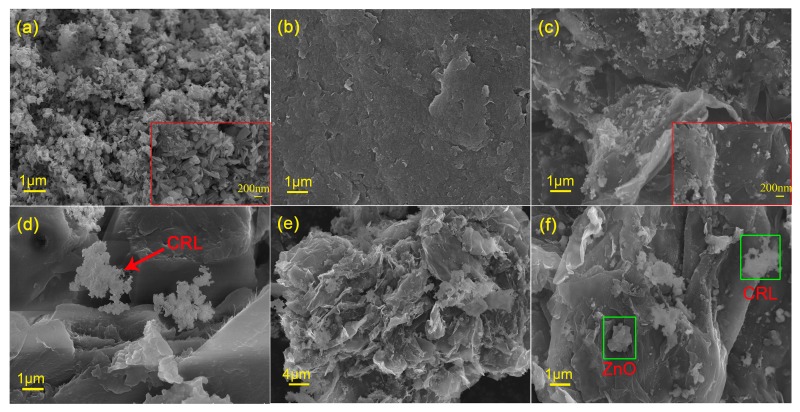
Scanning electron microcopy (SEM) images of (**a**) ZnO; (**b**) pristine GO; (**c**) GO/ZnO; (**d**) GO@CRL; (**e**,**f**) GO/ZnO@CRL.

In order to clarify the microstructure of the materials, the morphological features of the involved composites were examined via field-emission scanning electron microscopy. The agglomerated shape of the ZnO nanoparticleswhich synthesized by the typical method was clear and it can be seen that the composite microstructure was a blocky structure composed of nanoparticles with good uniformity (Figure 1a). The surface of the GO has uniform and smooth layers (Figure 1b), with most of the nanosheets stacked and entangled together. It wasas obvious that the formed GO/ZnO composites, as shown in Figure 1c, exhibited an exfoliated layered GO sheet structure with a few in-situ formed ZnO clustered particles decorated randomly inside or on the surface of the GO sheets, which prevent their effective restacking. Upon immobilization of lipase on the GO materials, as observed in reported literature [35,36], a sponge-like agglomerated structure appeared, which belong to the lipase attached on the sheets of GO surface (Figure 1d). The accumulation of aggregated protein should contribute to a high concentration of soluble protein during the immobilization process and may cause a mixture of different lipases in open and closed conformation. For GO/ZnO@CRL materials, a more exfoliated layered structure of graphene oxide sheets is observed (Figure 1e), with sponge-like CRL agglomerated structures attached on or inside the GO sheets. Figure 1f is a zoom in picture of Figure 1e, where CRL and ZnO nanoparticles can be clearly distinguished on the GO sheets, indicating that GO/ZnO composite is an ideal carrier for lipase immobilization via intermolecular interactions.

**Figure 2:**
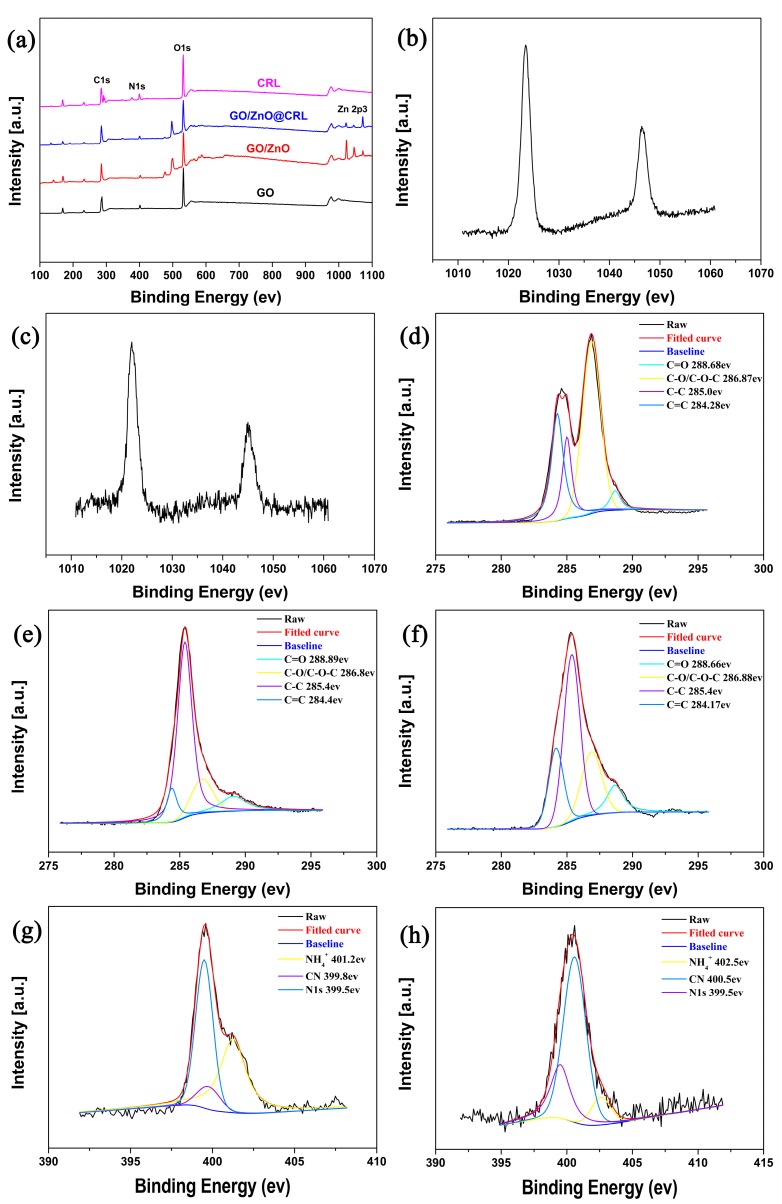
X-ray photoelectron spectroscopy (XPS) spectra scans; (**a**) wide scan XPS spectra of the supporting materials, high-resolution XPS spectra of Zn 2p scan (**b**) GO/ZnO, (**c**) GO/ZnO@CRL, C 1s spectra of (**d**) GO, (**e**) GO/ZnO and N 1s spectra of (**f**) CRL and (**g**) GO/ZnO@CRL.

X-ray photoelectron spectroscopy (XPS) was used to investigate the surface compositions and the valence states of these obtained materials (2). The wide scan XPS spectra of the samples (Figure 2a) displayed sharp peaks at the binding energies of 288 eV (C 1s), 400 (N 1s), 530 (O 1s) and 1023 eV (Zn 2p^3/2^) respectively, indicating the existence of C, O, N and Zn on the surface of corresponding materials. The high-resolution XPS spectra (Figure 2b,c) of the Zn 2p scan exhibited two peaks, located at 1023 eV (Zn 2p^3/2^) and 1046 eV (Zn 2p^1/2^). Deconvoluted peaks of GO (Figure 2d) are displayed at four positions corresponding to C=C sp^2^ (284.28 eV), C–C sp^3^ (285.00 eV), C–O and/or C–O–C (286.87 eV), and C=O (288.68 eV). The decorated ZnO nanoparticles on the surface of the GO sheet shifted the C–C sp^3^ peak in GO/ZnO (Figure 2e) to a higher binding energy by 0.39 eV and was accompanied by an increase of the intensity of the C=C sp^2^ peak in GO/ZnO compared to that in GO. In addition, the peaks of C–O and/or C–O–C in GO/ZnO are slightly decreased compared to those in GO. These results suggested that GO was partly reduced in the modified nanomaterials. The N 1s spectra of CRL and GO/ZnO@CRL are shown in Figure 2f,g. For CRL, the peaks at a binding energy of 399.50 eV, 399.77 eV and 401.25 eV were assigned to N 1s, C–N and NH^4+^, respectively. The peaks of C–N and NH^4+^ in GO/ZnO@CRL are obviously shifted to a higher binding energy by 0.83 and 1.35 eV, indicating that CRL was bound by electrostatic interactions. All the results from the XPS analysis demonstrate that the supporting materials were prepared successfully.

**Figure 3:**
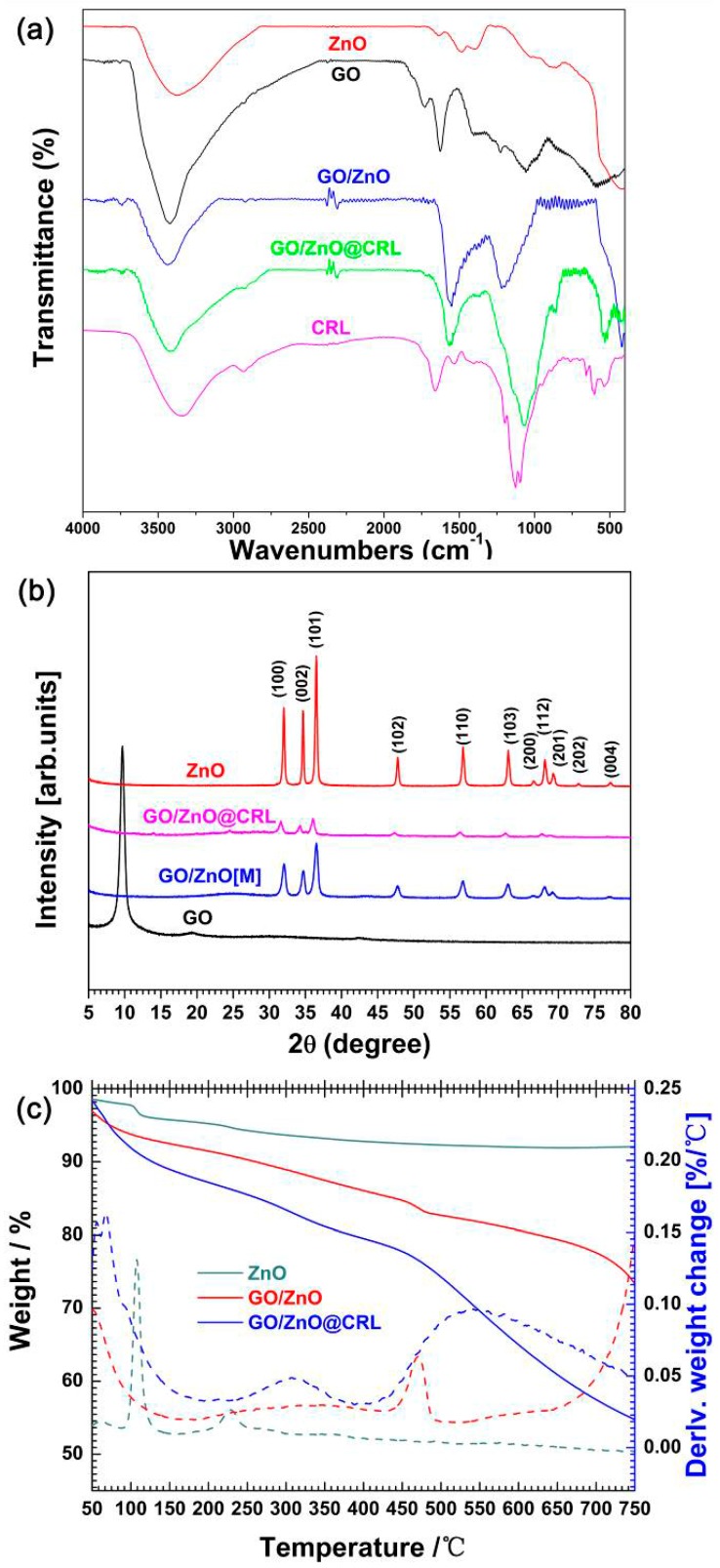
(**a**) The FT-IR spectra of GO, GO/ZnO, ZnO, CRL and GO/ZnO@CRL; (**b**) the XRD patterns of GO, ZnO, GO/ZnO and GO/ZnO@CRL; (**c**) the TG porfiles of GO/ZnO, ZnO and GO/ZnO@CRL.

The Fourier transform infrared spectroscopy FT-IR of pristine GO, CRL, ZnO, GO/ZnO, and GO/ZnO@CRL are shown in Figure 3a. The spectra of the pristine GO and ZnO are similar to the former literature results [37]. In the curve of pristine GO (c), the peaks at 1735 cm^−1^, 1624 cm^−1^, 1229 cm^−1^ and 1062 cm^−1^ correspond to the C=O stretching vibrations of the carbonyl and COOH groups located at the edges of the GO networks, C=C vibration of the skeleton, C–OH vibration and C–O stretching vibration of an epoxide groups, respectively. After decoration with ZnO nanoparticles, the C=O peak was not observed with the same intensity compared to the GO spectrum (c), suggesting that the GO sheet was partly reduced, which is consistent with the XPS results. Meanwhile, the intrinsic small peaks of pristine GO and ZnO, ranging from 1725 to 725 cm^−1^, were replaced by two new broad peaks appeared at 1550 cm^−1^ and 1210 cm^−1^. The peak at 1550 cm^−1^ is attributed to the skeleton vibrations of the benzene ring, caused by the reduction of GO, whereby the benzene ring conjugation was recovered. The 1210 cm^−1^ peak can be ascribed to the C–O binds of hydroxyls on the surface of GO. Furthermore, a significant decrease in the peak at 3410 cm^−1^ corresponding to the –OH stretching was observed, indicating an interaction between ZnO and the surface functional groups on GO, and suggesting that ZnO nanoparticles were grafted onto the GO sheets. Compared to the curve of CRL, the –OH peak at 3390 cm^−1^ was decreased when the enzyme was adsorbed onto GO/ZnO. These results suggest that the N–H bond of CRL interacts with the O–H bond of the supporting material via hydrogen bonding. On the other hand, the absorption at 3410 cm^−1^ which is characteristic of the stretching vibration of the primary amine (–NH_2_) group on the GO/ZnO@CRL materials was broadened and enhanced compared to the curve of GO/ZnO. Signals at 2964 and 2860 cm^−1^ for anti-symmetric and symmetric stretching vibrations of –CH_2_–, which was brought by the CRL, were observed, while, the strong broad peak at 1070 cm^−1^ appeared due to the influence of the stretching vibration absorption of C–O from CRL too. The above FT-IR results indicate that the supporting materials were prepared well, and the CRL has been immobilized on the GO/ZnO particles succesfully by intermolecular interactions.

Figure 3b shows typical XRD patterns of the as-prepared samples. For ZnO nanoparticles, the XRD patterns manifest predominant diffraction peaks at 2θ values 31.71°, 34.36°, 36.2°, 47.44°, 56.49°, 62.76°, 66.59°, 68.18°, 69.29°, 72.41° and 76.83°. These peaks are well matched with standard JCPDS card No. 36-1451. The curve of GO is also shown, where a unique characteristic peak was observed at a 2θ of 10.5°, which was assigned to the crystal plane (001). For GO/ZnO and GO/ZnO@CRL, their peaks were similar, and the characteristic diffraction peak of GO is not observed, which is caused by the reduction of GO, however, the characteristic peaks of ZnO do appear, revealing that after immobilization of the enzyme, the XRD profile is not changed, suggesting that the crystalline structure of the support remained almost unchanged after enzyme attachment. These results further indicate that GO/ZnO and GO/ZnO@CRL were prepared successfully.

The thermo-gravimetric (TG) analysis results of ZnO, GO/ZnO and GO/ZnO@CRL are given in Figure 3c. As can be seen from the figure, the ZnO particles show a remarkable thermostability over the whole weight loss of 7.88 wt %. A large mass loss (22.32%) of GO/ZnO is observed, especially around at 470 °C, which could be attributed to the decomposition and vaporization of various function groups present at different positions on GO. After immobilization of CRL, two weight loss peaks appeared that started at 300 °C and 485 °C with the decomposition of CRL and functional groups on GO, respectively, and an additional weight loss of about 29.02 wt % of GO/ZnO@CRL was observed when the temperature rose to 530 °C. The final weight loss from 620 °C to 750 °C might be due to the continuous decomposition of more stable carbon in GO. The TG analysis also proved the successful preparation of the GO/ZnO@CRL.

### 2.3. Relative Activity of the Immobilized CRL

**Figure 4:**
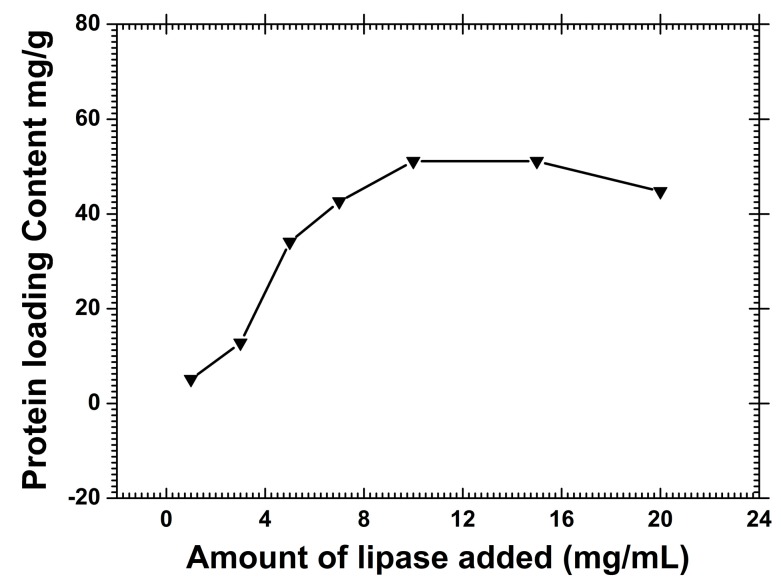
Effect of the amount of lipase added on protein loading content.

Furthermore, to examine the practical applicability of the GO/ZnO materials, maintaining the spatial structure and catalytic activity of enzyme, herein, CRL was immobilized on the GO/ZnO composites via a physical absorption method and the reaction conditions were examined. The efficiency of immobilization is expressed by the amount of lipase bound on a carrier of uniform mass, which was determined by the Bradford method. The results, shown in Figure 4, indicate that the protein loading content went up as the initial concentration of CRL solution increased, which is due to the abundance of suitable functional groups on the surface of GO/ZnO nanoparticles and the high specific surface area of GO. The protein loading reached the maximum when the initial concentration of CRL solution was 15 mg/mL, which was the optimum concentration for immobilization. Afterwards, the loading content of protein decreased as the amount of lipase solution further increased, which is probably due to the excess lipase coating affecting the physical properties of the support and reduced enzyme mobility [38]. The electrostatic repulsions between the same charges of CRL molecules led to less protein loading when abundant CRL was introduced in to the GO/ZnO particles. Finally, under the optimum reaction conditions, the lipase loading amount on the GO/ZnO nanoparticles was about 73.52 mg of protein per g.

**Figure 5:**
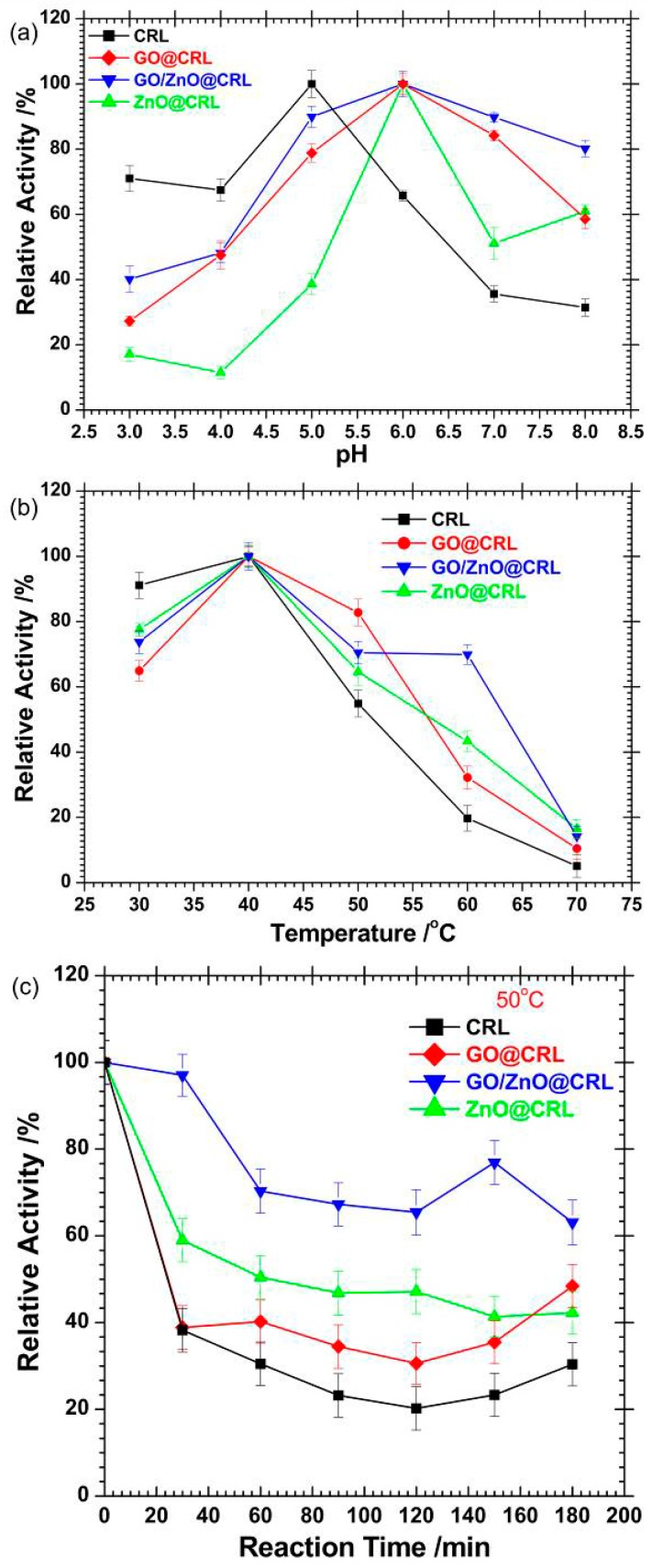
(**a**) Effects of the pH value on the activities of free and immobilized lipase in various pH (3.0–8.0) at 37 °C; (**b**) effects of the temperature on the activities of free and immobilized lipase at various temperatures (30–70 °C) at pH 7.0. The activity under optimal conditions was taken as control (100%); (**c**) thermal stabilities of free and immobilized lipase with various times (0–180 min) at pH 7.0 for 50 °C. The initial activity was defined 100%.

The pH and temperature are important parameters that influence the enzymatic activity in aqueous solution. For comparison simplicity, we assigned the maximum activity value under optimal conditions as 100% and the activities were expressed as relative activities under other conditions. The results are shown in Figure 5a. Similar to reported results, the optimum pH shifted from 5.0 for the free lipase to 6.0 for immobilized CRL, which is due to the proton micro-environment being influenced by the groups on the carriers when the free lipase directly accesses the substrate in the soluble form during reactions. As we know, the net charge of the protein is positive at pH values below the isoelectric point (pI) and negative at pH values above the pI. At the optimum pH of 6.0, the electrostatic interaction between CRL and carriers was enhanced due to the negatively charged CRL and the positively charged ZnO [34]. Meanwhile, the pH profiles of the immobilized CRL lipase showed a broader pH range, which demonstrated the improved stability in comparison to that of the free enzyme. At pH = 8.0, the relative activity of GO/ZnO@CRL can reach 81% of its initial activity, while the free CRL can only reach 32%, and the performance of GO@CRL and ZnO@CRL is also better than that of free CRL at a high pH. The remarkable pH tolerance of the immobilized CRL can be explained by the fact that the integrity of CRL was preserved over a wider pH range by immobilization on the obtained materials. Moreover, the pH profiles of the GO/ZnO@CRL are the broadest in the three immobilized lipases for the change of the micro-enviroment around CRL after immobilization [39]. Compared to free CRL, immobilized CRL has advantages in terms of stability at different pH values, which was similar to previous reports.

Temperature could also affect the relative activity of free CRL and immobilized CRL. As shown in Figure 5b, the optimum temperature for both free and immobilized lipases is 40 °C. GO/ZnO@CRL shows the best relative activity in the whole temperature range from 30 °C to 60 °C (71% of the initial activity at 60 °C). The relative activity of the free CRL decreased rapidly (19% of the initial activity at 60 °C) with an increase in temperature, due to denaturation. Lower temperature was adverse to the expression of the high activity of immobilized lipase, which may result from the hampered substrate penetration into the bound lipase [40].

In contrast, the three immobilized CRLs are more heat-resistant than the free form in the higher temperature region. This may be explained that the formation of hydrogen bonds and electrostatic interactions between lipase and supports that can restrict the conformation distortion or damage during temperature elevated.

The thermal stability of lipase is one of the most important application criteria for different applications [41,42]. From Figure 5c, we can see that both the free and immobilized CRL exhibit the similar trend: the residual activity declined along with the prolonged reaction time while the free CRL declined less and more slowly. The free CRL activity was reduced to 31% while GO/ZnO@CRL kept its activity at about 63% when the incubation time reached at 180 min. Meanwhile, the relative activity of the three immobilized lipases is considerably higher. In comparison, the thermal stability of GO/ZnO@CRL is much higher than that of the other two immobilized lipases. These results indicate that immobilized lipase is more stable than free one and GO/ZnO@CRL exhibited the highest thermal stability among the three obtained immobilized lipases. The enhancement of thermal stability is probably attributable to the strong interactions formed between the lipase and the carrier, which are beneficial to maintain a stable configuration of the immobilized lipase. GO/ZnO@CRL, with the synergetic effect of GO and ZnO nanoparticles, the optimized configuration and higher lipase loading amount that can be achieved, displayed the best performance.

**Figure 6:**
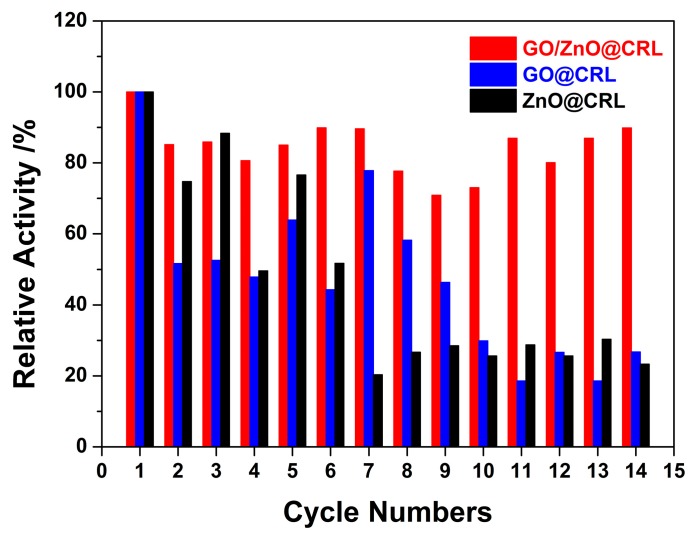
Reusability of the immobilized lipase. Reaction conditions: 30 mg GO/ZnO@CRL, 1 mL 0.5% *p*-NPP ethanol solution (*w*:*v*), 1 mL 0.1 M pH 7.0 phosphate buffer, 37 °C, 30 min. The biocatalyst was separated by centrifugation and the reusability of the immobilized preparation was monitored for 14 successive cycles. The activity determined on the first cycle was taken as the control (100%).

Reuse stability is one of the most important aspects for any potential industrial application. In this paper, the recyclability of the GO/ZnO@CRL was evaluated in consecutive batches of hydrolysis of *p*-nitrophenylpalmitate (*p*-NPP) carried out under identical reaction conditions as described previously. According to Figure 6, the three obtained immobilized lipases exhibit a different cycle performance. The GO/ZnO@CRL maintained the higher relative activity at 90% after 14 recycles, while lower relative activities of 28% and 23% were observed for GO@CRL and ZnO@CRL, respectively. For GO@CRL, the significant difference may be due to the properties of GO, whereby the GO@CRL materials was easily agglomerated leading to CRL active site being covered during the reaction. For ZnO@CRL, the strength of the interactions between CRL and ZnO is weak, which results in leakage of CRL from the support and bad reuse stability. After the ZnO nanoparticles decorated the GO, the excellent reuse stability of GO/ZnO@CRL can be explained by the following aspects: (i) the agglomeration of GO was greatly suppressed; (ii) the strength of the interaction between lipase and GO/ZnO was enhanced with the abundant functional groups on the surface of GO particles increased by the introduction of the hydroxyls of ZnO; (iii) the enhanced specific surface area of GO/ZnO provides a favorable reaction site and higher lipase loading amount for catalysis. In addition, the solvent may cause the leaching of the enzyme, responsible of the lower activity in the recycling process. Interestingly, a slightly fluctuation of activity in the three obtained immobilized lipase was observed, which might be because the activity is influenced provisionally due to partial lipase present inside the layers within the curly GO sheets during the reaction [3].

## 3. Materials and Methods

### 3.1. Materials

Graphene flakes were purchased from Sigma-Aldrich (St. Louis, MO, USA), and used without further purification. *Candida rugosa* lipase (lyophilized powder, Type VII, 700 U/mg solid) and *p*-nitrophenylpalmitate (*p*-NPP) were also purchased from Sigma-Aldrich. Phosphate buffer solution (PBS, 0.1 M, pH = 7.0), which was prepared by mixing standard stock solution of 0.1 M KH_2_PO_4_ and 0.1 M K_2_HPO_4_, was used as the supporting electrolyte. Unless otherwise stated, reagents were of analytical grade and used as received.

### 3.2. Preparation of GO

Graphene oxide (GO) was prepared using a modified Hummer method [22]. Graphite flakes (0.6 g), and NaNO_3_ (3.0 g) were mixed with an ice-water bath, then H_2_SO_4_ (120 mL) and KMnO_4_ (16.0 g) was added successively and the mixture stirred slowly for 2 h. After the reaction, the flask was placed in a thermostatted water bath, and stirred at 35 °C for 30 min. Then the mixture was cooled down in ice-water bath and deionized water was added gradually. Then, one more time, the flask was put into the thermostatted water bath, and stirred for 20 min at 98 °C. The mixture was allowed to cool down to room temperature. A bright yellow solution was obtained after H_2_O_2_ (250 mL, 30%) was added, the precipitate was collected by centrifugation and washed with dilute hydrochloric acid. Finally, the precipitate was washed with double distilled water until the pH of the supernatant was neutral.

### 3.3. Synthesis of ZnO and GO/ZnO Materials

The GO/ZnO composites were prepared by a simple hydrothermal method. Firstly, GO (0.5 g) was added to distilled water (50 mL) to form a homogeneous dispersion, and then Zn(CH_3_COO)_2_·2H_2_O (0.578 g) was added. The mixture was sonicated for 1 h, and then NaOH (1.053 g), ethylene glycol (10.53 mL) and sodium citrate (5.0 g) were added and stirred quickly. After stirring for 1 h, the mixture was transferred into a Teflon-lined stainless steel autoclave and reacted at 100 °C for 12 h. The obtained samples were thoroughly washed with ethanol and water and dried in a vacuum oven at 60 °C to obtain GO/ZnO materials. For the synthesis of nanostructured ZnO, the solution method was used, wherein Zn(CH_3_COO)_2_·2H_2_O (8.78 g) was added to a 4 M aqueous solution of sodium hydroxide, stirred well and refluxed at 90 °C for 1 h. After refluxing, the precipitate (a white powder) was neutralized with methanol and dried at room temperature.

### 3.4. Immobilization of Lipase

The obtained GO/ZnO, ZnO particles and GO were dispersed in lipase solution, respectively, and the three mixed solution was incubated at 37 °C in a shaker operating at 160 rpm for 3 h. The three mixture was washed with phosphate buffer (0.1 M, pH = 7.0) three times and then freeze dried in a vacuum chamber. The three obtained immobilized lipases were stored at 4 °C until used. The influences of initial concentration of CRL solution on efficiency of protein loading was explored by contacting GO/ZnO carrier (50 mg) with 1–20 mg/mL of CRL solution with different initial concentrations at a fixed temperature of 37 °C and pH 7.0 for 3 h.

### 3.5. Lipase Activity Assay

The protein concentration was determined by the Bradford method, using BSA as the standard. The experimental result showed that the free *Candida rugosa* lipase solution contained 0.062 mg of protein per mL. The amount of immobilized lipase was calculated by detecting the amount of un-immobilized lipase. The activities of the free and immobilized lipases were measured by the hydrolysis of *p*-NPP in 0.1 M PBS at pH 7.0 and 37 °C (ε = 1805 M^−1^ cm^−1^ under these conditions) for 5 min under 160 rpm. The concentration of the *p*-nitrophenol (*p*-NP) hydrolysis product was measured using a spectrophotometric method at 410 nm. The blank of hydrolysis of *p*-NPP under similar conditions without protein was also measured and the relative results are shown in Supplementary Materials. One unit of activity (U) was defined as the amount of enzyme that hydrolyzes 1 μmol of *p*-NPP per minute under the conditions described previously.

The effect of temperature and pH on the activity were investigated by incubating the free and immobilized lipase at different temperatures and pHs for 30 min, respectively. Thermal stability assays were studied by measuring the residual activities of the lipase after incubation from 0 h to 3 h at 50 °C [40]. The reusability of the immobilized lipase was determined by the following procedure: 30 mg of immobilized lipase was used for hydrolysis of *p*-NPP solution in phosphate buffer for 30 min at 37 °C under mild stirring (90 rpm). After each batch reaction, the immobilized lipase was washed three times with absolute ethanol in order to remove the product, *p*-NP, and dried with a nitrogen purge. Four cycles were performed during one day, and the immobilized lipase was stored at room temperature overnight. The lipase activity was measured as the above description. Similar to the previous reports [43,44], both of the free and immobilization lipase remain active during testing procedure.

### 3.6. Characterization

The X-ray diffraction (XRD) spectra were collected using a D8 ADVANCE X-ray diffractometer (Bruker, Karlsruhe, Germany). The morphological images of the products were obtained on a SU8010 field emission scanning electron microscope (Hitachi, Tokyo, Japan). A TENSOR27 instrument (Bruker, Karlsruhe, Germany) were used to record the Fourier transform infrared spectroscopy (FTIR) spectra. The thermal stability of samples was studied with a thermogravimetry (TG) analyzer (TAQ50, Netzsch, Selb, Germany) at a heating rate of 50 °C min^−1^ in a nitrogen atmosphere. XPS analysis of the surface was conducted with a PHI Quantera II X-ray photoelectron spectroscope (ULVAC-PHI, Japan) equipped with an Al Kα X-ray radiation source.

## 4. Conclusions

In summary, three support materials—GO, ZnO and GO/ZnO—were prepared and relevant characterization was carried out to systematically study their influence on the performance of lipase immobilization. *Candida rugosa* lipase (CRL) was immobilized on the supports via physical absorption with a maximum protein loading amount of 73.52 mg·g^−1^. All the performance features of the immobilized CRL are enhanced, while GO/ZnO supported materials exhibit superior performance than free CRL. The high specific surface area and excellent mechanical properties of the supports facilitate the ordering of catalytic reactions and the long-term operational stability and thermal stability of lipase. Free CRL, GO@CRL and ZnO@CRL lost 64%, 62% and 41% of their initial activity, respectively, within 30 min at 50 °C, while GO/ZnO@CRL still maintained 63% of its initial activity even after 180 min at 50 °C. Furthermore, the GO/ZnO@CRL shows excellent recycling performance, whereby 90% of the original activity is maintained after 14 reuse cycles. Based on the results obtained from this study, we propose that GO/ZnO@CRL is a promising biocatalyst for practical applications.

## Supplementary Materials

Supplementary materials are available online.
