# Supplementary material for: Preparation of Carriers Based on ZnO Nanoparticles Decorated on Graphene Oxide (GO) Nanosheets for Efficient Immobilization of Lipase from Candida rugosa
Source: Molecules. 2017 Jul 19;22(7):1205. doi: 10.3390/molecules22071205 (PMC6152098; doi:10.3390/molecules22071205)
Supplement: Supplementary file 1 [file molecules-22-01205-s001.pdf]

Supporting Information

Preparation of carriers based on ZnO nanoparticles decorated on graphene oxide (GO) nanosheets for efficient immobilization of lipase from Candida rugosa

Shan Zhang, Jie Shi *, Qianchun Deng, Mingming Zheng, Chuyun Wan, Chang Zheng, Ya Li and Fenghong Huang *

The experiment of the the blank of hydrolysis of pNPP at these conditions without protein was measured as follow:
1 mL of 0.5% *p*-NPP ethanol solution (w/v) and 1 mL of 0.1 M PBS (pH 7.0), then incubated at 37 °C for 5 min. The result showed in fig.1 and proved that the hydrolysis of *p*-NPP without CRL existed extremely minimum proportion (~2%) when regarded the hydrolysis of *p*-NPP with CRL as 100%. We considered that the influence of hydrolysis of *p*-NPP can be negligible.

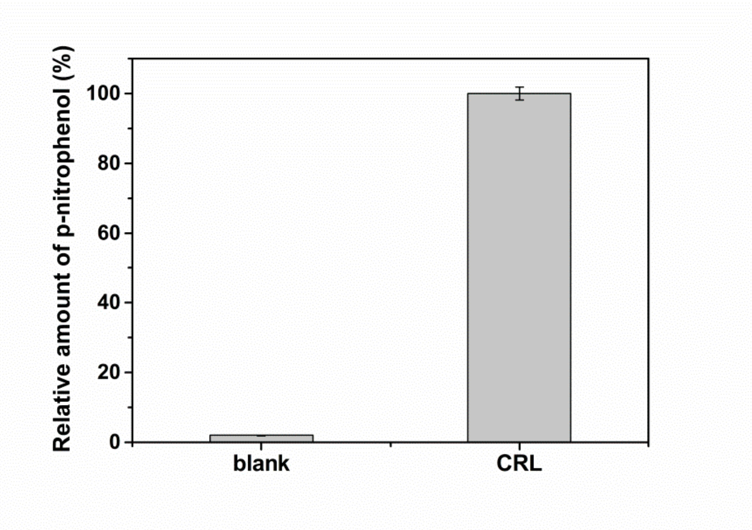

Figure S1. Relative amount of p-nitrophenol in the hydrolysis of *p*-NPP without and with CRL. Conditions: 1 mL of 0.5% *p*-NPP ethanol solution (w/v), 1 mL of 0.1 M PBS (pH 7.0), incubated at 37 ℃ for 5 min.
